# Feasibility and Acceptability of an Early Childhood Obesity Prevention Intervention: Results from the Healthy Homes, Healthy Families Pilot Study

**DOI:** 10.1155/2014/378501

**Published:** 2014-10-27

**Authors:** Akilah Dulin Keita, Patricia M. Risica, Kelli L. Drenner, Ingrid Adams, Gemma Gorham, Kim M. Gans

**Affiliations:** ^1^Institute for Community Health Promotion, Department of Behavioral and Social Sciences, Brown University, P.O. Box G-S121-8, Providence, RI 02908, USA; ^2^Kinesiology & Health Science, Stephen F. Austin State University, P.O. Box 13015 SFA Station, Nacogdoches, TX 75962, USA; ^3^School of Human Environmental Sciences, University of Kentucky, Lexington, KY 40506, USA; ^4^Center for Health, Intervention, and Prevention, Department of Human Development and Family Studies, University of Connecticut, 348 Mansfield Road, Unit 1058, Room 320, Storrs, CT 06269, USA

## Abstract

*Background.* This study examined the feasibility and acceptability of a home-based early childhood obesity prevention intervention designed to empower low-income racially/ethnically diverse parents to modify their children's health behaviors. *Methods.* We used a prospective design with pre-/posttest evaluation of 50 parent-child pairs (children aged 2 to 5 years) to examine potential changes in dietary, physical activity, and sedentary behaviors among children at baseline and four-month follow-up. *Results.* 39 (78%) parent-child pairs completed evaluation data at 4-month follow-up. Vegetable intake among children significantly increased at follow-up (0.54 cups at 4 months compared to 0.28 cups at baseline, *P* = 0.001) and ounces of fruit juice decreased at follow-up (11.9 ounces at 4 months compared to 16.0 ounces at baseline, *P* = 0.036). Sedentary behaviors also improved. Children significantly decreased time spent watching TV on weekdays (*P* < 0.01) and also reduced weekend TV time. In addition, the number of homes with TV sets in the child's bedroom also decreased (*P* < 0.0013). *Conclusions.* The findings indicate that a home-based early childhood obesity prevention intervention is feasible, acceptable and demonstrates short-term effects on dietary and sedentary behaviors of low-income racially/ethnically diverse children.

## 1. Introduction

Childhood obesity remains a significant public health concern. While national health and nutrition examination survey (NHANES) reports suggest that obesity may have declined among children aged 2–5 years [1], these beneficial declines have not been evidenced across all geographic regions, racial/ethnic groups, or income levels [1, 2]. Recent data from the 2008–2011 Pediatric Nutrition Surveillance System found that while there were significant decreases in obesity prevalence among low-income preschoolers in 19 US states/territories, there were no significant changes in 21 US states/territories and there were significant increases in three US states/territories [2]. Further, research findings indicate that since 2008 there have been no appreciable changes in obesity trends among low-income preschoolers in the US state of Rhode Island, with obesity prevalence remaining above 16 percent [2]. Thus, Rhode Island remains one of the US states/territories with the highest obesity prevalence among low-income preschoolers for the 43 reporting US states/territories.

Additional findings from NHANES demonstrate that since 2003 there have been no changes in childhood obesity overall [1, 3]. In fact, one-third of children remain overweight or obese; 17% are obese [1] and severe obesity (≥ Class 2 adult obesity) is increasing with 8% of children meeting the criteria [3]. This is cause for concern because childhood obesity is associated with severe obesity in adulthood, early onset of obesity-related comorbidities such as metabolic syndrome, type 2 diabetes, cardiovascular diseases (CVD), certain cancers, negative impacts on mental health and quality of life, and increased economic and medical costs [2, 4, 5]. Recent estimates suggest that relative to a normal weight 10-year-old child the direct lifetime incremental medical cost for an obese 10-year-old is $12,660; in the aggregate, this will account for $9.4 billion in medical costs for this age group alone [4]. Taken together, the available evidence underscores the critical need to increase our efforts to reduce childhood obesity particularly in early life and prevent and/or delay concomitant onset of obesity-related comorbidities, the negative impacts on quality of life, and the economic consequences.

Childhood obesity is particularly prevalent among low-income children, as well as African American and Latino children [1, 6, 7], which suggests that it is essential to develop focused, appropriate, and targeted intervention strategies in these populations [7, 8]. The prevention and treatment of overweight in youth hinges on helping children and their families develop new lifestyles and create supportive environments in which healthful eating and physical activity (PA) can be promoted [9–11]. Family-based interventions are effective in the treatment of childhood obesity [12], but most of these interventions have been time intensive and costly and therefore not sustainable or scalable after research funding ends [13]. Moreover, most have focused mainly on nonminority, middle, or high income families and older children. Thus, there is a pressing need to develop and test early childhood obesity prevention and treatment approaches for low-income and minority families that are effective but also practical, acceptable, and sustainable [8, 9].

The Institute of Medicine strongly recommends that obesity prevention intervention begins in early childhood [14] and focus on prevention efforts among children from birth to five years. This is a critical age range because the mean age at which obesity begins is 5.5 years [15–17] and BMI at age 8 is predicted by BMI at age 2 [18]. Additionally, evidence suggests that children's eating and physical activity behavioral patterns are established in early life and are more difficult to change after the age of 5 [19–21]. Intervention research findings indicate that attempts to induce children to change their food preferences are more effective with younger than older children [8]. This suggests that interventions should target younger children to prevent obesity and to help achieve the US Task Force on Childhood Obesity goal of reducing childhood obesity prevalence to 5% by 2030 [22].

Modifying the home/family environment and parent behaviors are crucial intervention components for the prevention of early childhood obesity [23]. Family environments are vital for the development of food preferences, patterns of food intake, and eating styles that shape children's weight status [24]. Parents play an important role in shaping early eating patterns in children by controlling availability and accessibility of foods, meal structure, and food socialization practices. Parent related behaviors including food-related parenting style, modelling healthful eating behaviors, encouraging physical activity, and/or discouraging sedentary behaviors convey values and attitudes that promote children's health through reinforcing specific behaviors [12, 25]. Additionally, intensive involvement of parents in interventions to change overweight children's dietary and PA behaviors contributes to long term weight maintenance [12, 25]. When interventions change parental behavior toward children, children's behaviors change correspondingly, even if the child is not directly involved in the intervention [12]. In fact, greater weight loss and higher consumption of healthy foods are achieved with parent-focused interventions compared with interventions in which children are the main agents of change [12].

Although there has been considerable growth in the number of childhood obesity prevention interventions with parents of preschool age children in a variety of settings [26–29], more intervention efforts are needed. The results from these previous interventions demonstrate that parent-focused, childhood obesity prevention interventions are feasible and effective in creating some healthy behavior changes and outcomes among both parents and preschool age children [26–28, 30]. One such intervention, the fit women, infants and children (Fit WIC) pilot program, was implemented in five US states with low-income ethnically diverse parents [31, 32] and children who participated in the US federal program, special supplemental nutrition program for women, infants and children (WIC). Results from one of the Fit WIC pilot programs found that parents made significant changes in health behaviors and increased family fitness-related activities [32]. Further, research findings from another Fit WIC program found that parents increased self-efficacy to limit children's TV viewing, reduced actual TV time for both parents and children, and increased physical activity among children [33]. Other studies that focus on changing parent behaviors in the home setting also have found significantly less engagement in restrictive parental feeding practices among low-income Native American parents [27] and less aversion to mealtime among preschoolers, less weight gain, and lower BMIs among both children and parents [34]. Additional intervention studies also report increased availability of fruits and vegetables in the home and increased parent role modelling of fruit and vegetable intake with concomitant increases in children's intakes [35]. However, more childhood obesity prevention interventions are needed that (1) build upon promising results of these previous studies, [26, 30] (2) combine multiple health behaviors (i.e., physical activity, sedentary behavior, and dietary components), (3) engage low-income and ethnically diverse parents, (4) focus on the home environment, (5) include tailored intervention materials, (6) incorporate effective counseling methods, and (7) use less costly intervention methods that could be more easily replicated.

Thus, the purpose of this intervention, healthy homes, healthy families (HHHF), is to address existing gaps in the literature by conducting a pilot feasibility and acceptability study of a parent-driven, home-based intervention to modify health lifestyle behaviors among low-income racially/ethnically diverse children aged 2 to 5 years. The findings from HHHF will inform the design and implementation of a future randomized controlled trial.

## 2. Methods

### 2.1. Study Design

HHHF was an early childhood obesity intervention designed to encourage parents to improve healthy lifestyle behaviors related to eating and physical activity for themselves and their children. The study design was a prospective design with pretest/posttest measurement that combined telephone surveys and in-home visit measures collected at baseline and 4-month follow-up with 50 parent-child pairs. The study received approval from the Brown University Institutional Review Board. All participants received a financial incentive upon completion of each study visit.

### 2.2. Eligibility and Recruitment

The study recruitment occurred from 2009 to 2012, at twelve special supplemental nutrition program for women, infants and children (WIC) offices in low-income communities in Rhode Island. The research assistant approached WIC clients in the waiting room to tell them about the study and to ask if they would be interested in participating.

Interested participants were screened for eligibility. Study inclusion criteria required that participants were a parent or legal guardian of a child who was 2 to 5 years of age at the date of the baseline survey and had an age-sex specific body mass index (BMI) of 50th percentile or greater. The adult needed to be 18 years of age or older, live with the child at least 75 percent of the time, speak and read English, and be knowledgeable about the child's diet and physical activity behaviors. Eligible participants were asked to complete a baseline phone survey administered by trained interviewers using a computer automated telephone interface (CATI) system. Upon completion, research assistants were scheduled to visit the home at the convenience of the participant parent and child to complete an in-person survey, anthropometric measures, and a home audit. Verbal informed consent was received for the baseline telephone survey and then written informed consent (and verbal assent for children aged 4 and over) was received at the home visit. Upon completion of the home visit, parent-child pairs were considered enrolled. This process was repeated four months later as a follow-up assessment.

### 2.3. Intervention

HHHF included four sets of tailored written materials, three brief motivational interviewing (MI) telephone calls delivered by a trained lay counselor, a physical activity video tailored to the child's age, and a TV time monitoring device (TV Allowance by MINDMASTER, INC) to help parents monitor/restrict child's time spent on TV.

#### 2.3.1. Theoretical Framework

**Figure 1:**
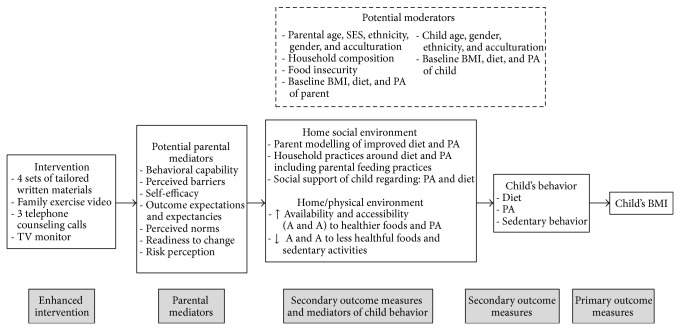
Healthy homes, healthy families intervention logic model.

The intervention was informed by social cognitive theory (SCT) [36–38], the conceptual model described by Golan and Weizman [39] and focus groups with the target audience and WIC nutrition counselors. The HHHF framework emphasized a familial approach to the prevention and treatment of overweight in young children with parents as the primary agent of change. As recommended by the Expert Panel of the Maternal and Child Health Bureau of Health Resources and Services Administration and Department of Health and Human Services, HHHF emphasized healthy lifestyle changes and no weight reduction [40]. HHHF focused on the formulation of new norms for healthy eating within the family through parents as role models and as sources of authority. HHHF also incorporated facilitating parental cognitive and behavioral change, increasing parenting skills and environmental change [39]. The HHHF intervention logic model is presented in Figure 1.

SCT is based on reciprocal determinism where a person's behavior, personal factors, and the environment interact constantly and where change in one domain affects changes in the other two domains [36–38]. Three major constructs of the SCT, self-regulation (personal regulation of goal-directed behavior), behavioral capability (knowledge and skills to perform specific tasks), and self-efficacy (confidence in one's ability to perform a particular behavior or overcome barriers to the behavior) were applied to HHHF intervention development. HHHF promoted self-regulation and outcome expectations through both the tailored intervention materials and the motivational interviewing (MI) components. Parents had the opportunity to choose topics for each mailing from a list of primary target behaviors that were an issue for their family. This provided opportunities for self-monitoring, decision making, and problem solving. The tailored written materials supported behavioral capability by providing the information needed for parents to modify the behaviors found to be associated with diet and PA in children and families. Parents' self-efficacy was developed by providing opportunities for them to choose to get materials to help them overcome specific barriers that they were experiencing. The MI calls offered social support and further developed self-efficacy through the exploration of desires, abilities, reasons, and needs for change [41, 42]. Counselors elicited positive outcome expectancies (benefits of change), encouraged problem solving if parents discussed barriers, and asked parents what steps they would take in the direction of change (goal setting).

#### 2.3.2. Materials

After the baseline home assessment, study staff installed the TV monitor on the TV that the child used most often. Since the primary goal of the TV monitor was as an intervention tool to increase parents' self-efficacy for setting TV restrictions and limiting the child's time spent watching TV, we did not collect any data from this device. Approximately 1-2 weeks later, participants received their first package of tailored written intervention materials. The tailored written materials were mailed out in four stages over a 20-week period (approximately every 4 weeks), and the lay counselor MI calls occurred approximately 2 weeks after the mailing of each set of materials. A final set of tailored materials were mailed 1-2 weeks after the final counseling phone call. Materials were microtailored (tailored messages embedded into a page) or macrotailored (entire pages chosen or not). We accomplished the tailoring by using algorithms based on parents' answers to survey questions and home audit results as well as parent choice. We generated tailored feedback reports for each family on all target child behaviors, the home environment, and parent role modelling behaviors. We also personalized materials with the participant's and child's name.

The tailored printed materials focused on eight target behaviors found to be associated with obesity in children and families. These behaviors (increasing fruits and vegetables, reducing sugary drinks, limiting juice, low-fat instead of high fat milk, increasing physical activity, limiting fast food, removing TV from the child's bedroom, and limiting screen time) were all within control of the parent. If the family was not meeting the guideline for a target behavior, the computer populated a list of choices. We then presented the list to parents as areas where change was possible. Parents then chose a topic for each mailing from this list of primary target behaviors that were an issue for their family. We conducted a similar process for barriers that parents identified as problem areas such as the cost of healthy eating, cost of physical activity, children upset about changing foods or household rules, picky eaters, time for healthy eating, time for PA, children's choices/habits, lack of knowledge/skill, and lack of social support. Parents could receive up to a total of five barriers pages. In addition, parents could choose up to four tailored recipe pages.

#### 2.3.3. Motivational Interviewing-Based Telephone Intervention

In between each of the four tailored mailings, parents received a brief motivational interviewing (MI) call designed to support their efforts to make changes to the social and physical home environment [41, 42]. The MI calls were designed to be 10–20 minutes long and to be delivered three times over the course of the intervention. These calls were digitally recorded.

We recruited four women to serve as lay MI counselors for the enrolled parents/guardians (one dropped out early due to the time commitment). We selected counselors who resided in Rhode Island and who had some experience with behavior change interventions but not specifically with MI. One counselor was Hispanic and three were non-Hispanic white and all had experience working with low-income populations. A facilitator, Dr. Drenner, trained through the motivational interviewing network of trainers (MINT), trained the lay counselors over seven evenings for a total of 12 hours. The MI training focused on the primary principles and techniques of the overall MI style and also on how these elements related to the specific behavior change targets of HHHF.

Once the telephone counseling began, Dr. Drenner monitored a random sample of the recorded telephone counseling sessions and continued coaching the counselors in group meetings and in individual sessions. She held group coaching meetings approximately biweekly both in-person and via conference call. Additionally, she held individual coaching sessions via telephone that focused on feedback on one or more of the digitally recorded telephone calls. Coaching was an opportunity for counselors to get consultation on both the content of the calls and specific behaviors related to MI. Dr. Drenner coded random counselor telephone calls using elements of the Motivational Interviewing Treatment Integrity Scale on global scores of empathy, behavior counts of reflections, and open and closed questions [41, 43].

Intervention adherence assessment included counselor's focus on (1) a specific target behavior, (2) assessment of importance and confidence of the chosen behavior, (4) goal setting, and (5) on calls 2 and 3, checking with the parent to see if they had met the set goal. Counselors elicited parents' own desire, ability, reason, and need for change and self-efficacy for change through reflection and affirmation of parents' effort to create a healthy environment for their child and family. Each participant received a tailored MI feedback page in the subsequent mailing summarizing the importance and confidence regarding the topic they discussed as well as the next step that the participant said they would take. If the counselor was unable to complete the call (after 3 phone call attempts), the participant received an MI feedback page informing them of the missed call as well as when they would receive the next call and a set of tailored materials based on the last contact.

#### 2.3.4. Measures

*Anthropometrics.* Children's and parent's/guardian's heights and weights were measured at baseline and follow-up. To obtain height measurements, children were measured without shoes using a portable stadiometer (Seca 213). Height was measured to the nearest 0.1 cm and averaged across 2 measurements. To obtain weight measurements, children wore light clothing and were weighed without shoes to the nearest 0.1 kg using a digital scale (Tanita BWB-800S Digital Scale). The average of 2 weight measurements was taken. BMI was calculated using the formula kg/m^2^, from which the BMI for age-sex specific percentiles was calculated using the centers for disease control and prevention (CDC) 2000 growth charts.

*Dietary Habits.* At the time of study implementation, there was not a well validated dietary assessment tool for preschoolers that comprehensively assessed children's intake of the foods and beverages we were trying to change; so we modified questions on existing validated tools to be appropriate for asking parents about their child's intake. To assess the child's fruit, vegetable, sugar sweetened beverage, and soda intakes, we adapted items from the validated National Cancer Institute (NCI) fruit and vegetable all-day screener which measures participants' usual consumption over the past month. The all-day screener was validated by conducting cognitive interviews with adults and examining correlations of the measure with four nonconsecutive 24-hour dietary recalls (*r* = 0.50) [44]. To determine frequency of food/beverage intake, the original survey asked the following: “over the last month, how often did you drink/eat [item]?” There were 10 response options ranging from never to 5 or more times per day. To assess portion size, the survey asked the following: “each time you drank/ate [item], how much did you usually drink/eat?” Response options corresponded to the frequency and portion size of the respective food/beverage. For the HHHF study, we substituted each statement with “Your Child” instead of “You” so that we could assess children's intakes [44]. We also modified these portion size choices to be appropriate for amounts that a preschooler would consume using the MY Plate recommendations for young children [45, 46].

We also obtained questions used in the Fit WIC [32] study to assess parent reports of their children's water, milk, and 100 percent fruit juice intakes and children's frequency of eating at fast food restaurants [32]. These items were not validated but were modified from existing child-based questionnaires to be appropriate for preschool age children [32]. These questions have also been recommended for inclusion in national surveillance data collection by the New South Wales Centre for Public Health Nutrition in Australia [47].

*Physical Activity.* We assessed children's outdoor playing time using a validated measure developed by Burdette and colleagues for preschool children's activity [48]. The correlation of the outdoor play measure with accelerometer data was *r* = 0.20 [48]. Parents reported the time (in minutes) that children engaged in weekday outdoor activity and weekend outdoor activity.

*Sedentary Behaviors.* Parents reported children's TV use including the number of hours of TV/video/DVD/playing the child “usually watches” on weekdays and weekend days [49]. We also asked parents to report whether the child watches TV during meals and snacks. These questions have demonstrated high test-retest reliability (*r* = 0.94) with older children [50] and have been used successfully in studies with children 1–5 years of age [49–51].

*Parent Behaviors.* We assessed parent behaviors related to role modelling, the home food environment, family support for PA, family encouragement for PA/diet, and parent household rules related to PA/diet. To examine parent role modelling, we adapted items from the Home Environment Survey developed by Gattshall et al. [52]. We modified these items to align with HHHF outcomes based on results from in-depth cognitive interviews with HHHF parents. Example items include “on how many days last week did your child see you walk to get from place-to-place instead of drive?” and “on how many days last week, did your child see you eating fast food?” To examine the home food environment, we developed items specific to HHHF intervention outcomes including the number of times per day the parent provided the child with fruits and vegetables, the number of days per week the child consumed low-fat milk, and the number of days per week that healthy/unhealthy foods were available (See Table 3).

We examined parental support for child physical activity using three items from the Aventuras Para Niños study to inquire about parents/family activity together and transportation [53]. Response options ranged from 1 to 7 days per week. We also included a separate item about family support for the child to play outside that was developed specifically for this study. We also adapted items from the Aventuras Para Niños study [54] that examined whether parents provided praise/encouragement for children's diet and physical activity behaviors; we also created additional questions that were adapted to HHHF outcomes. Example questions included “on how many days this past week did you praise your child for eating fruits and vegetables” and “on how many days this week did you praise your child for being physically active.” We also examined parents' household rules related to diet/PA using items adapted from the Aventuras Para Niños Study and items developed specifically to the HHHF intervention outcomes [53, 54]. Based on pretest results from the cognitive interviews with HHHF participants, we modified the items and response options from the Aventuras Para Niños Study. Sample items include “how often do you limit the amount of time your child spends watching TV or videos” and “how often do you limit the amount of 100% fruit juice your child drinks.” Response options ranged from 1 = never to 5 = always.

*Demographics.* Parents self-reported parent and child gender, race, ethnicity, and age. Parents also self-reported marital status and socioeconomic status-related variables including employment, education, and total annual household income. Additional parent-reported demographics included household composition and food insecurity (i.e., how often the parent worried about having enough food in the home).

### 2.4. Data Analysis

Demographic variables were collected for parent, as well as the child, and categorized as follows: gender (male versus female), race (White, Black, Asian, Native Hawaiian or other Pacific Island, American Indian or Alaska Native, mixed race, other), and ethnicity (Hispanic versus non-Hispanic). Mean age and BMI were determined and treated as continuous variables. Descriptive statistics were computed with frequencies and proportions for categorical variables and means for continuous variables. Chi square tests were used to compare categorical psychosocial data and categorical demographic variables. General linear models were constructed to compare mean differences of dietary intake, physical activity, sedentary behaviors, child BMI, and parent behaviors pre-/posttest. Significance criterion was set at *α* < 0.05. All analyses were performed using SAS version 9.3 (SAS Institute, Cary, NC).

## 3. Results

**Figure 2:**
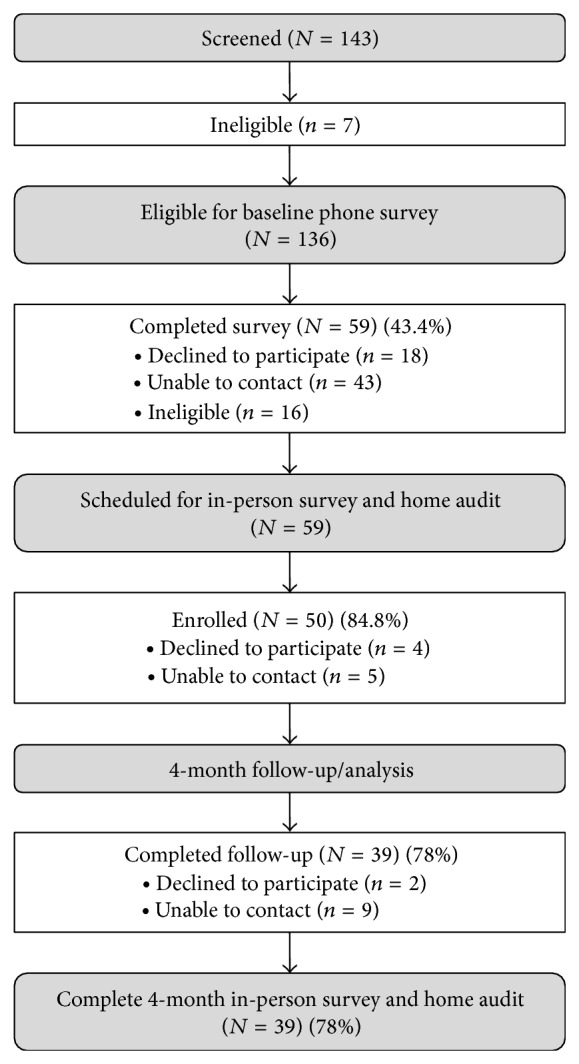
Healthy homes, healthy families intervention recruitment flow diagram.

Figure 2 presents the study recruitment flow diagram. Of the 143 potential child-parent pairs initially recruited by the research assistant, 7 were ineligible to complete additional screening. A total of 136 parent-child pairs were eligible to complete the phone survey; 59 completed the survey, 18 declined to participate, 43 were unable to be contacted, and 16 were ineligible to continue the screening process. Fifty-nine eligible parent-child pairs scheduled the in-person survey and home audit. At this stage, 4 declined to participate and 5 were unable to be contacted leaving a total of 50 parent-child pairs who enrolled in the intervention. At four-month follow-up, 39 parent-child pairs (78%) completed both the telephone and the home audit components of the evaluation, 2 declined to participate, and 9 were unable to be contacted.

Baseline demographic and BMI characteristics of the participating children and parents/guardians are presented in Table 1. Children enrolled averaged 3 years, 7 months of age, with parents/guardians averaging 31 years. All of the adult participants were parents and 98% of them were women. Forty percent of the parents described themselves as Hispanic, with 50% of the enrolled children being described as Hispanic. Almost half (48%) of the parents were White, 14% Black, and 4% mixed race and 38% of children were White, 14% Black, and 14% mixed race. Just over half (54%) of the parents were single, 36% were married, and the remaining 10% reported that they were separated or divorced. About one-quarter each of the participating parents were employed full time, part time, or unemployed, with an additional 12% homemakers, 10% students, and 4% disabled. The educational level attained for participating parents/guardians reached high school or general educational development (GED) credential for the highest proportion (46%) and some college or an associate's degree for 32%. The remaining group included those with less than high school education (8%), technical or vocational school (6%), and either a bachelors (6%) or postgraduate degree (2%). Just over one-third of the families had no other children in the home, but roughly a quarter each reported one or two children and 16% reported 3 or more other children in the home. Also, over one-third of parents/guardians were the sole adult at home, with 42% reporting two adults and 10% reporting three or more. Slightly more than one-fourth of parents/guardians reported food insecurity (concern over having enough food). Household income was generally low: 14% of parent-child pairs resided in households with <$6,000 per year and 20% in $6–$11,999 per year. Only 4% of parent-child pairs resided in households where the total annual income was between $24–$29,999 and 14% in the $36,000 or higher income group.

The average BMI of the children enrolled in HHHF was at the 65th percentile for age and sex. The recruited children were mostly within the range of 50th–85th percentile (72%), with an additional 14% each in the overweight (≥85th, <95th %ile) and obese (≥95th %ile) categories. The parents/guardians averaged a BMI of 29 kg/m^2^. The highest proportion of adult participants were obese (48% with BMI ≥ 30), 20% were overweight (BMI ≥ 25, <30), 28% were normal weight, and 4% were underweight.

### 3.1. Process Evaluation

Process evaluation measures are presented in Table 2. According to parent reports on the follow-up survey, over 72% of parents received three MI calls, 19% received two calls, 2.7% received no calls, and 5.5% reported other. However, according to process evaluation data from the counselors, fewer calls were completed; 16% of parents received three calls, 50% received two calls, 26% received one call, and 8% received no calls.

Parents/guardians reported that the health coach made them “think about their child's health a lot” (69%) and “felt understood by the health coach a lot” (74%). A very high proportion of parents/guardians agreed a lot that “they felt respected” (92%), that “the health coach expressed caring and understanding when discussing their child's health” (89%), and that “the health coach made it comfortable for [the parent] to talk about their child's health” (87%). Also, the parents/guardians agreed a lot that “the health coach addressed concerns about the child's health” (84%), “helped [the parent] to think about why health changes might be important to the child” (77%), and “helped [the parent] to set goals for positive change in the child's life” (71%).

Most parents reported receiving three (45%) or four (42%) mailings, and the majority read all or most of them (82%). Most parents found the materials somewhat (34%) or very (55%) interesting and 95% reported that “they were very clearly written.” Parents agreed a lot that “the materials were easy to read” (95%), “had information they could use,” (82%) or believe (71%), and “were written especially for [the parent]” (68%). At the time of the four-month follow-up, 87% were still using the written materials and 71% had shared the materials with others.

The TV monitor received somewhat mixed results. The monitor was used always or often (33%), sometimes (13%), but also rarely or never (35%), or the parents/guardians chose not to have a TV monitor (18%). Most parents/guardians (74%) agreed a lot that “the device was easy to use.” However, only about half of participating parents/guardians agreed a lot that “the monitor was useful” (43%) and “was a great tool because they could set it and forget it” (57%) and that “the device helped the child spend more time doing physically active things” (48%). Most parents disagreed a lot (52%) or a little (9%) that “the child would get upset when the TV monitor was turned on.”

### 3.2. Intervention Outcome Evaluation

Baseline and change in child outcome measures are presented in Table 3. At baseline, parents reported that children consumed 0.28 cups of vegetables and 0.96 cups of fruit each day. Also, children consumed a mean of 16 ounces of 100% fruit juice and 8.8 ounces of sweetened drinks per day. Children also averaged 14 ounces of water and 15 ounces of milk consumption per day. Parents also reported children eating fast food just over one time per week. Parents reported that children engaged in physical activity and averaged 195 minutes on week days and 206 minutes on weekends. Also, children averaged 97 minutes on week days and 136 minutes on weekends of outside play. Conversely, children also engaged in sedentary behaviors and averaged 147 minutes on week days and 149 minutes on weekend days watching TV.

Although mean BMI percentile did decrease (−1.77 kg/m^2^) from baseline to month 4, this change was not significant. However, significant change was found in children's daily vegetable intake. Higher intake was reported at month 4 of follow-up (0.54 cups) compared with baseline (0.28 cups, *P* = 0.001). In addition, significant reductions were observed in mean ounces of fruit juice consumed each day (11.94 ounces at 4 months compared to 16.01 ounces at baseline, *P* = 0.036).

While there were no significant changes in intakes for other beverages, all changes were in the direction expected with 4-ounce reductions in sweetened beverage intakes per day and a 0.6-ounce increase in water intake per day. Also, the reduction in the number of times in which children consume fast food each week approached statistical significance (*P* = 0.09). Physical activity and sedentary behaviors also changed over the course of the intervention. From baseline to 4 months, reported minutes of time spent playing outside significantly decreased (97 minutes on weekdays and 136 minutes on weekend days at baseline compared to 60 minutes on weekdays and 71 minutes on weekend days at 4 months *P* = 0.0243 weekday; *P* = 0.0082 weekend). However, parents/guardians reported that children spent less time watching TV on weekdays (111 minutes compared with 147 minutes per day at baseline, *P* < 0.01); weekend TV time also decreased by 20 minutes, but this change was not statistically different. We also observed that the percent of households with TVs present in the child's bedroom significantly decreased from 70% to 60%, *P* < 0.0013 from baseline to follow-up (data not shown in table).

Baseline and 4-month change in parent behaviors related to parent role modelling, the home food environment, family support for PA, family encouragement for PA/diet, and parent household rules are presented in Table 4. Parent role modelling: parents reported significant increases in the days that their child saw them drink low-fat milk (0.87 days, *P* = 0.0324) and there was a borderline significant decrease in the number of days that their child saw them eating fast food (−0.33 days, *P* = 0.0513). Parents reported statistically significant increases in the number of days that their child saw them walking from place-to-place (0.71, *P* = 0.0292) and exercising (0.72, *P* = 0.0094). Parents also reported a statistically significant decrease in the average minutes per day that their child saw them watching TV (−47.18 min, *P* = 0.0158). The home food environment: parents reported an increase in the number of times that they gave their child 1% or skim milk (1.13 times, *P* = 0.0350). Family support for PA: there were no significant changes in family support for PA from baseline to four-month follow-up. Family encouragement for PA/diet: parents at follow-up were more likely to praise their child for drinking low-fat milk (2.2 days compared with 0.9 days per week, *P* = 0.0181) and for eating fruits and vegetables (4.5 days compared with 2.6 days per week, *P* < 0.0001). Also, parents were more likely after the intervention to encourage their child to watch less TV (4.3 days compared to 2.4 days per week, *P* = 0.0105). Parent household rules: most parents at follow-up were more likely to limit the number of days that their child spent playing video games (3.51 days compared to 3.22 days, *P* = 0.0271). Also, parents were more likely to limit the number of days that their child drank 100% juice (3.15 days compared to 2.5 days, *P* = 0.0017) and limit the number of days that their child ate fast food (4.44 days compared to 3.82 days, *P* = 0.0099).

## 4. Discussion

The main objective of this study was to examine the feasibility of a home-based early childhood obesity intervention to modify parent and child health behaviors. This pilot intervention showed great promise in demonstrating that a home-based intervention could be successful in changing some parental behaviors as well as dietary and sedentary behaviors of children. Many changes were either statistically significant or in the posited direction, which is impressive given that the sample size was only 50 parent-child pairs and the intervention was monthly for only four months in duration. Overall, participating parents/guardians reported positively on the components of the intervention. The telephone counselors were well received and the tailored written materials were well used. While there were some discrepancies in parent reports of receipt of MI counseling calls, we think this could be due to the parents confusing the counseling calls with the baseline and follow-up evaluation calls or confusing attempts to reach them with actual MI calls. However, the response to the TV monitor was somewhat mixed; though some parents/guardians seemed to fully use the device others did not report using it at all. The overall pilot feasibility, intervention findings, and parent reported acceptability demonstrate significant potential for HHHF to be implemented as a future randomized controlled trial for the prevention of childhood obesity. Additionally, we had good participant retention at four-month follow-up.

The current study also found significant improvements in children's daily servings of vegetables and reductions in 100% juice intake, but no statistically significant changes in sweetened beverage, water, milk intake, or fast food consumption were evidenced. On average, children's total servings of vegetables almost doubled over the course of the intervention. However, these intake levels are still lower than recommendations for children of this age (1 to 1.5 cups each of fruits and vegetables per day) [45]. Many of the other dietary changes, especially reductions in sweetened beverage and fast food intake, might have been statistically significant with a larger sample size. Research findings from other early childhood interventions and systematic review studies also found that increases in fruit and/or vegetable intake were key behavioral changes made but that there were no changes made in sweetened beverage intake or fast food consumption [30, 35, 55, 56]. In contrast, results from the ROMP & Chomp community-wide intervention with young children in Geelong, Australia, found both significant reductions in nutrient-poor energy dense foods and sugar sweetened beverage intakes and also increased fruit, vegetable, and water intakes [21].

It is important to note that more than one-fourth of HHHF participating parents identified food insecurity as a key concern, which may have affected intervention efficacy. The finding of high levels of parent reported food insecurity is similar to reports from other interventions with low-income parents of young children [9]. The HHHF intervention did include practical strategies for low-resource households such as choosing produce that is in season and healthy options for frozen or canned products. However, future interventions should continue to acknowledge the resource limitations of low-income ethnically diverse households by strengthening these components further. Additional practical strategies that might improve intervention efficacy for low-resource households might include community gardening [57] and bonus buck programs for farmers markets [58].

Contrary to our hypotheses, we found unexpected declines in parent reports of children's outdoor playing time on both weekdays and weekend days. On average, parents reported that children participated in one hour less of total daily outdoor physical activity at the four-month follow-up assessment. These findings are disconcerting because early childhood physical activity patterns track into adulthood and high levels of physical activity in early childhood mitigate physical activity declines evidenced during adolescence [20]. The National Association for Sport and Physical Education recommends that young children (birth to age of 5) engage in 120 minutes of daily physical activity with 60 minutes of structured and 60 minutes of unstructured physical activity [59]. Parents in HHHF reported that children were physically active between 164 and 206 minutes per day. While other research findings suggest that, in the US, the majority of young children meet the daily recommendations [20, 60–62], we think the estimates from the HHHF parents are likely overestimates. Parents made anecdotal comments like “my child is hyper,” and we think that they may have misjudged physical activity for motion. Other studies have also found that parents overestimate children's physical activity [63, 64]. For example, Corder et al. found that 80 percent of parents in an obesity prevention study in San Diego, California, overestimated their child's physical activity [64].

We also tested the hypothesis that seasonality may have influenced changes in outdoor physical activity from baseline to four months. There were no significant differences in baseline physical activity (weekday or weekend) between summer/early fall relative to fall/winter group participants. Also, seasonality did not significantly affect changes in weekday outdoor physical activity (*P* = 0.238). The lack of significance for weekday activity may have been mitigated by daycare/school recess and outdoor physical activity polices. However, participants who were assessed at four-month follow-up during the late fall experienced significantly larger declines in minutes of weekend outdoor physical activity relative to the group who was assessed at four-month follow-up during the winter months (−71.8 minutes relative to −12 minutes, *P* = 0.408). It is also possible that the significant reductions in physical activity observed over the course of the intervention were due to the timing (seasonality), but other explanations could also include initial overreporting of physical activity by parents and realization of this overreporting after participating in the intervention. These findings suggest that the future interventions should devote more efforts to preserving and/or increasing children's physical activity levels, especially on weekend days. In addition, future research with families of young children should not rely on self-reports but instead use objective measures such as accelerometry and give parents tailored feedback on the real activity patterns of their children.

Regarding children's sedentary behaviors, time spent watching television was significantly reduced during the weekday and somewhat declined on weekend days. Children decreased their weekday television time by almost 50 minutes from baseline to four-month follow-up but did not decrease TV time as much on weekend days. This significant reduction in TV screen time resulted in children meeting the guidelines recommended by The American Academy of Pediatrics [65] of no more than two hours of TV per day. However, there are some limitations as parents self-reported the data. Future studies might consider the use of television monitors to objectively measure whether TV use decreases [66, 67]. Research findings indicate that parental attitudes, norms, and parental screen time as well as having a television in the child's bedroom are all risk factors for increased screen time among young children [68]. Future interventions should modify these parent related behaviors and additional research should examine parent's qualitative reports to better understand the decision making processes that parents use for screen time on weekdays versus weekends.

The findings from HHHF provide mixed support for changes in parent behaviors associated with children's health behaviors. The study demonstrates favorable improvements in some of the parent behaviors related to parent role modelling, the home food environment, family encouragement for PA/diet, and household rules. Contrary to other intervention results [32, 35] however, HHHF participants did not make any significant changes in modelling of fruit and vegetable intakes or time spent being physically active with children as a family. Despite not making changes in many dietary practices, HHHF participants did report increases in role modelling of physical activity behaviors and decreases in modelling of sedentary behaviors. These results may suggest that HHHF parents felt more confident in making PA related changes than dietary changes. Future interventions with similar populations should direct more efforts to increasing parent role modelling of dietary changes and actual intakes of fruits and vegetables. Although findings are equivocal, many of the changes in parent behaviors are consistent with systematic review studies that suggest that effective parent-driven childhood obesity interventions for preschool age children incorporate behavior change strategies that are predicated on behavioral theories and include restructuring of the home environment [26, 28].

### 4.1. Limitations

While informative, this study is not without some limitations. The study recruited children at all levels of obesity risk, which included many children at a healthy weight and potentially more motivated parents/guardians. Additionally, as this was a pilot intervention and was underpowered to detect differences in key outcomes, the sample size was small and effect size estimates with small samples have large standard errors and wide confidence intervals. The pretest/posttest design was a limitation which might have affected the validity and generalizability of study findings [69]. This study did not include a control group, so some of the changes seen could have been due to factors other than the intervention. Also, there were no follow-up measures administered past the posttest intervention assessment so we were unable to examine whether changes were maintained over time. However, this study found significant improvements in many health behaviors related to obesity and many behavioral changes operated in the posited direction. Additionally, many of the parent related behaviors were significantly changed suggesting that the intervention favorably improved behaviors within the parents' control. Future randomized trials should be conducted with a control or comparison group to be able to assess the real effect sizes of the intervention and additional follow-up assessments to determine whether behavioral changes made during the intervention are maintained over time.

Despite the limitations, this study has a number of strengths and is one of few home-based early childhood obesity prevention interventions specifically designed for low-income diverse racial/ethnic populations. This study recruited directly from WIC clinics, thus ensuring recruitment of families who were eligible to receive income based support from federal programs. The sample was predominantly low-income and ethnically and racially diverse thus reaching populations who are at significantly higher risk for future obesity and related comorbidities. There was also good participant retention at four-month follow-up. Additionally, the goals of this intervention were aligned with current recommendations and focused on changing health behaviors for the long term instead of weight loss.

### 4.2. Conclusions and Next Steps

HHHF was a parent-driven home-based intervention that incorporated tailored written materials and video, nutrition information, and MI along with TV monitors and an age-matched children's exercise video. This intervention appeared to be effective in changing some aspects of children's behavior and their home environments through changes made by parents. However, a randomized trial is necessary to truly test the efficacy of this intervention. Such trial will be planned in the near future. We will also analyze correlates of children's BMI, diet, PA, and sedentary behavior as well as predictors of change, which will aid in future intervention development. Furthermore, to broaden the reach of the intervention to a larger population, we would like to be able to offer the intervention in Spanish as well as English. It may also be worthwhile to test other channels in addition to print mailings for providing tailored messages, that is, tailored video, internet, text messaging, smart phones, and etcetera. It might also be interesting to study the effectiveness of combining a home-based intervention like HHHF with a pediatric health care provider intervention or an intervention in child care settings.

## Figures and Tables

**Table 1:** Demographic characteristics of the 50 parent-child pairs in healthy homes, healthy families.

Variable	Mean ± SD or *n* (%)
Parent gender (female)	49 (98)
Relationship to child	
Mother	49 (98)
Father	1 (2)
Percent Hispanic or Latino? (parent)	20 (40)
Percent Hispanic or Latino? (child)	25 (50)
Race (parent)	24 (48)
White	7 (14)
Black	1 (2)
Asian	1 (2)
Native Hawaiian or other Pacific Island	3 (6)
American Indian or Alaska Native	4 (2)
Mixed race	12 (24)
Other	24 (48)
Race (child)	
White	19 (38)
Black	7 (14)
Asian	1 (2)
Native Hawaiian or other Pacific Island	1 (2)
American Indian or Alaska Native	3 (6)
Mixed race	7 (14)
Other	12 (24)
Marital status	
Single	27 (54)
Married	18 (36)
Divorced	1 (2)
Separated	4 (8)
Employment status	
Employed full time	12 (24)
Employed part time	13 (26)
Unemployed	12 (24)
Disabled	2 (4)
Student	5 (10)
Homemaker	6 (12)
Education	
Less than high school	4 (8)
High school or general educational development credential (GED)	26 (46)
Vocational or technical school or Some college or associate degree	19 (38)
Bachelor's degree	3 (6)
Postgraduate degree	1 (2)
Number of other children living in household	
0	17 (34)
1	13 (26)
2	12 (24)
3	5 (10)
4	2 (4)
5	1 (2)
Number of adults (including yourself) living in household	
1	18 (36)
2	21 (42)
3	6 (12)
4	3 (6)
5	1 (2)
6	1 (2)
Worried about not having enough food (yes)	14 (28)
Annual household income	
<$6,000	7 (14)
$6,000 to $11,999	10 (20)
$12,000 to $17,999	4 (8)
$18,000 to $23,999	10 (20)
$24,000 to $29,999	5 (10)
$30,000 to $35,999	2 (4)
$36,000	7 (14)
Don't know or refused	5 (10)
BMI category (parent)	
Underweight	4 (2)
Healthy weight	14 (28)
Overweight	10 (20)
Obese	24 (48)
BMI category (child)	
Underweight (<5th percentile)	0
Within range (5th to <85th percentile)	36 (72)
Overweight (85th to <95th percentile)	7 (14)
Obese (≥95th percentile)	7 (14)
Mean Age	
Parent or guardian	28.38 ± 6.18
Child (age in months)	43.12 ± 11.88
Mean BMI	
Parent or guardian	29.81 ± 8.21
Mean BMI %ile for age and sex	
Child	65.36 ± 27.48

**Table 2:** Process evaluation data.

Variable	*n* (%)	*n* (%)	*n* (%)	*n* (%)	*n* (%)
Health coach overall					

	None	One	Two	Three	Other

How many phone calls did you receive from the health coach (reported by participants)	0 (0)	1 (2.78)	7 (19.44)	26 (72.22)	2 (5.56)
Actual calls completed according to counselors	4 (8)	13 (26)	25 (50)	8 (16)	

	Not at all	A little bit	Some	A lot	

How much did the health coach make you think about your child's health	1 (2.56)	2 (5.13)	9 (23.08)	27 (69.23)	
How much did you feel understood by the health coach	0 (0)	1 (2.56)	9 (23.08)	29 (74.36)	

	Agree a lot	Agree a little	Neither agree nor disagree	Disagree a little	Disagree a lot

*The health coach *					
Made it comfortable for me to talk about my child's health	34 (87.18)	1 (2.56)	4 (10.26)	0 (0)	0 (0)
Respected me	36 (92.31)	0 (0)	3 (7.69)	0 (0)	0 (0)
Helped me to think about why health changes might be important to my child	30 (76.92)	4 (10.26)	3 (7.69)	1 (2.56)	1 (2.56)
Expressed caring and understanding when talking with me about my child's health	35 (89.74)	1 (2.56)	2 (5.13)	0 (0)	1 (2.56)
Addressed my concerns about my child's health	33 (84.62)	2 (5.13)	2 (5.13)	1 (2.56)	1 (2.56)
Helped me to set a goal for positive changes in my child's health	28 (71.79)	7 (17.95)	3 (7.69)	1 (2.56)	0 (0)
I felt pressured by the health coach to make changes in my child's health	2 (5.13)	0 (0)	3 (7.69)	2 (5.13)	32 (82.05)
*The HHHF materials *					
Were written specifically for you	26 (68.42)	4 (10.53)	5 (13.16)	3 (7.89)	0 (0)
Had information you could use	31 (81.58)	4 (10.53)	1 (2.63)	1 (2.63)	1 (2.63)
Had information you could believe	27 (71.05)	8 (21.05)	3 (7.89)	0 (0)	0 (0)
Were easy to read	36 (94.74)	2 (5.26)	0 (0)	0 (0)	0 (0)
*The TV monitor *					
Was easy to use	17 (73.91)	2 (8.70)	0 (0)	1 (4.35)	3 (13.04)
Was useful	10 (43.48)	4 (17.39)	2 (8.70)	2 (8.70)	5 (21.74)
Is a great tool for parents because it is a “set it and forget it” device for them	13 (56.52)	4 (17.39)	3 (13.04)	1 (4.35)	2 (8.70)
Helped your child spend more time doing physically active things	11 (47.83)	2 (8.70)	4 (17.39)	2 (8.70)	4 (17.39)

**Table 3:** Changes in child outcomes from baseline to month 4 and change scores for healthy homes, healthy families participants.

Variable	BL intervention group mean ± Std. Dev (95% CI) (*n*)	M4 intervention group mean ± Std. Dev (95% CI) (*n*)	Change (BL to M4) intervention group mean ± Std. Dev (95% CI) (*n*)	*P* value
*BMI *				
Child BMI for age	65.36 ± 27.48 (57.55–73.17) (50)	63.82 ± 29.73 (54.19–73.46) (39)	−1.77 ± 10.93 (−5.31–1.78) (39)	0.319
*Food habits *				
Servings of vegetables/day	0.28 ± 0.34 (0.19–0.38) (50)	0.54 ± 0.64 (0.33–0.75) (39)	0.28 ± 0.53 (0.11–0.45) (39)	0.001∗
Servings of fruit/day	0.96 ± 1.13 (0.64–1.28) (50)	1.17 ± 1.17 (0.78–1.56) (37)	0.21 ± 1.04 (−0.13–0.56) (37)	0.222
Ounces of 100% fruit juice/day	16.01 ± 15.10 (11.72–20.30) (50)	11.94 ± 11.14 (8.33–15.55) (39)	−3.92 ± 11.27 (−7.57–−0.27) (39)	0.036∗
Ounces of sweetened drinks and soda/day	8.80 ± 18.52 (3.48–14.12) (49)	5.06 ± 12.77 (0.86–9.25) (38)	−4.23 ± 19.65 (−10.78–2.32) (37)	0.198
Oz/day child drinks water	13.98 ± 13.47 (10.77–17.85) (49)	13.42 ± 8.52 (10.62–16.22) (38)	0.61 ± 9.31 (−2.46–3.67) (38)	0.691
Oz/day child drinks milk	15.40 ± 9.78 (12.62–18.18) (50)	13.44 ± 6.88 (11.21–15.67) (39)	−0.46 ± 8.20 (−3.12–2.20) (39)	0.727
Times/week child eats fast food	1.16 ± 1.23 (0.81–1.51) (50)	0.86 ± 0.83 (0.59–1.13) (39)	−0.29 ± 1.06 (−0.64–0.05) (39)	0.091
*Physical activity habits (min) *				
Weekday child exercises	194.98 ± 171.56 (145.70–244.26) (49)	164.21 ± 170.42 (108.20–220.23) (38)	−13.35 ± 138.86 (−59.65–32.95) (37)	0.562
Weekend day child exercises	206.02 ± 185.71 (152.68–259.36) (49)	182.90 ± 169.28 (128.02–237.77) (39)	5.74 ± 132.50 (−37.82–49.29) (38)	0.791
Weekday child spends playing outside	96.80 ± 107.49 (66.25–127.35) (50)	59.51 ± 58.14 (40.66–78.36) (39)	−22.28 ± 59.33 (−41.52–−3.05) (39)	0.024∗
Weekend day child spends playing outside	136.40 ± 126.76 (100.37–172.43) (50)	70.67 ± 73.91 (46.71–94.62) (39)	−41.13 ± 91.99 (−70.95–−11.31) (39)	0.008∗
*Sedentary behavior (min) *				
Weekday child spends watching TV	146.90 ± 98.71 (118.85–174.95) (50)	110.77 ± 81.19 (84.45–137.09) (39)	−49.87 ± 99.88 (−82.25–−17.49) (39)	0.003∗
Weekend day child spends watching TV	149.00 ± 96.27 (121.64–176.36) (50)	133.72 ± 91.16 (104.17–163.27) (39)	−20.38 ± 119.80 (−59.22–18.45) (39)	0.294

^*^Indicates significant group differences, *P* < 0.05.

**Table 4:** Changes in parent behaviors from baseline to month 4 and change scores for healthy homes, healthy families participants.

Variable	BL Mean ± Std. Dev (*n*) (95% CI)	M4 Mean ± Std. Dev (*n*) (95% CI)	Change BL to M4 Mean ± Std. Dev (*n*) (95% CI)	*P* value (2 sided)
*Parent role modelling of food practices *				
Times/day child saw you eat fruit or vegetables w/meal	2.44 ± 2.03 (50) (1.86–3.02)	2.42 ± 2.05 (38) (1.75–3.09)	0.11 ± 3.22 (38) (−0.95–1.16)	0.8414
Times/day child saw you eat fruit or vegetables as a snack	1.90 ± 2.01 (50) (1.33–2.47)	1.77 ± 1.51 (39) (1.28–2.26)	−0.03 ± 2.42 (39) (−0.81–0.76)	0.9476
Days child saw you drink low-fat milk	1.74 ± 2.62 (50) (1.00–2.48)	2.41 ± 2.90 (39) (1.47–3.35)	0.87 ± 2.45 (39) (0.08–1.67)	0.0324∗
Days child saw you eating fast food	1.30 ± 1.37 (50) (0.91–1.69)	0.90 ± 1.33 (39) (0.47–1.33)	−0.33 ± 1.03 (39) (–0.67–0)	0.0513
Times/day child saw you drink sweetened drinks	1.84 ± 1.60 (50) (1.39–2.29)	1.49 ± 1.32 (39) (1.06–1.91)	−0.15 ± 1.91 (39) (–0.77–0.47)	0.6184
*Parent role modelling of activity practices *				
Days child saw you walk from place to place	1.41 ± 2.21 (49) (0.77–2.04)	1.77 ± 2.38 (39) (1.00–2.54)	0.71 ± 1.93 (38) (0.08–1.34)	0.0292∗
Days child saw you exercising	0.73 ± 1.45 (49) (0.32–1.15)	1.54 ± 2.16 (39) (0.84–2.24)	0.72 ± 1.64 (39) (0.19–1.25)	0.0094∗
Min/day child saw you watching TV	131.90 ± 100.58 (50) (103.31–160.49)	88.08 ± 58.62 (39) (69.07–107.08)	−47.18 ± 116.61 (39) (−84.98–−9.38)	0.0158∗
Min/day child saw you playing on computer	73.60 ± 109.44 (50) (42.50–104.70)	50.38 ± 77.75 (39) (25.18–75.59)	−17.05 ± 68.86 (39) (−39.37–5.27)	0.1303
*Parental support for child's physical activity *				
Days you did physically active things w/your child	2.20 ± 2.15 (50) (1.59–2.81)	2.46 ± 2.17 (39) (1.76–3.17)	0.28 ± 2.65 (39) (−0.58–1.14)	0.5095
Days you did physically active things as a family	1.80 ± 1.82 (50) (1.28–2.32)	1.33 ± 1.80 (39) (0.75–1.92)	−0.41 ± 2.05 (39) (−1.07–0.25)	0.2187
Days/week you took child to be physically active	3.40 ± 2.09 (50) (2.81–3.99)	2.97 ± 2.24 (39) (2.25–3.70)	−0.38 ± 3.01 (39) (−1.36–0.59)	0.4305
Days/week you suggested child to play outside	3.46 ± 2.62 (50) (2.72–4.20)	2.44 ± 2.01 (39) (1.78–3.09)	−0.79 ± 3.06 (39) (−1.79–0.20)	0.1133
*Home food environment *				
Times/day you gave child fruit to eat	1.86 ± 1.22 (49) (1.51–2.21)	2.21 ± 1.49 (39) (1.72–2.69)	0.47 ± 1.62 (38) (−0.06–1.01)	0.0802
Times/day you gave child vegetables to eat	1.78 ± 1.52 (50) (1.35–2.21)	1.64 ± 1.22 (39) (1.24–2.04)	−0.05 ± 1.69 (39) (−0.60–0.49)	0.8503
Days/week you have cut up fv for child to eat	3.88 ± 2.60 (50) (3.14–4.62)	3.81 ± 2.22 (32) (3.01–4.61)	0.22 ± 2.71 (32) (−0.76–1.20)	0.6510
Days per week the child consumed low-fat milk	3.84 ± 3.21 (50) (2.93–4.75)	4.64 ± 3.14 (39) (3.62–5.66)	1.13 ± 3.22 (39) (0.08–2.17)	0.0350∗
Days/week had soda in your home for child to drink	0.82 ± 1.84 (50) (0.30–1.34)	0.90 ± 1.70 (39) (0.35–1.45)	−0.05 ± 2.03 (39) (−0.71–0.61)	0.8752
Days/week you had sweetened drinks in your home for child to drink	2.90 ± 2.87 (50) (2.08–3.72)	2.85 ± 2.87 (39) (1.92–3.78)	−0.31 ± 3.13 (39) (−1.32–0.71)	0.5429
Days/week you had sweets for child to eat	3.94 ± 2.67 (50) (3.18–4.70)	3.74 ± 2.59 (39) (2.90–4.58)	−0.64 ± 2.99 (39) (−1.61–0.33)	0.1881
Days/week you had salty snack for child to eat	2.88 ± 2.50 (50) (2.17–3.59)	3.36 ± 2.45 (39) (2.56–4.15)	0.13 ± 2.68 (39) (−0.74–1.00)	0.7665
*Parent praise/encouragement for diet and/PA *				
Days/week you praised child for drinking low-fat milk	0.92 ± 2.13 (50) (0.32–1.52)	2.23 ± 3.14 (39) (1.21–3.25)	1.26 ± 3.18 (39) (0.23–2.29)	0.0181∗
Days/week you praised child for eating fv	2.63 ± 2.58 (49) (1.89–3.37)	4.51 ± 2.58 (39) (3.68–5.35)	1.85 ± 2.42 (39) (1.06–2.63)	<0.0001∗
Days/week you praised child for not drinking sweetened drinks	1.06 ± 2.26 (49) (0.41–1.71)	1.51 ± 2.43 (39) (0.73–2.30)	0.53 ± 2.48 (38) (−0.29–1.34)	0.1988
Days/week you encouraged child to watch less TV	2.40 ± 2.60 (50) (1.66–3.14)	4.31 ± 2.24 (26) (3.40–5.21)	1.35 ± 2.48 (26) (0.34–2.35)	0.0105∗
Days/week you praised child for being physically active	2.88 ± 2.90 (49) (2.05–3.71)	3.54 ± 2.78 (39) (2.64–4.44)	0.49 ± 2.99 (39) (−0.48–1.46)	0.3153
*Parent household rules *				
Limit number of days child spends watching TV/videos	3.22 ± 1.28 (50) (2.86–3.58)	3.51 ± 1.32 (39) (3.09–3.94)	0.46 ± 1.25 (39) (0.06–0.87)	0.0271∗
Limit number of days child plays video games	4.48 ± 1.76 (50) (3.98–4.98)	4.56 ± 1.70 (39) (4.01–5.11)	0.31 ± 1.70 (39) (−0.24–0.86)	0.2665
Limit number of days child spends on computer	4.54 ± 1.80 (50) (4.03–5.00)	5.00 ± 1.54 (39) (4.50–5.50)	0.56 ± 1.94 (39) (−0.07–1.19)	0.0779
Limit number of days child drinks 100% juice	2.50 ± 1.39 (50) (2.11–2.89)	3.15 ± 1.44 (39) (2.69–3.62)	0.62 ± 1.14 (39) (0.25–0.98)	0.0017
Limit number of days child eats fast food	3.82 ± 1.22 (50) (3.47–4.17)	4.44 ± 1.05 (39) (4.10–4.78)	0.69 ± 1.59 (39) (0.18–1.21)	0.0099

^*^Indicates significant group differences, *P* < 0.05.
